# Artificial Neural Networks in the Outcome Prediction of Adjustable Gastric Banding in Obese Women

**DOI:** 10.1371/journal.pone.0013624

**Published:** 2010-10-27

**Authors:** Paolo Piaggi, Chita Lippi, Paola Fierabracci, Margherita Maffei, Alba Calderone, Mauro Mauri, Marco Anselmino, Giovanni Battista Cassano, Paolo Vitti, Aldo Pinchera, Alberto Landi, Ferruccio Santini

**Affiliations:** 1 Department of Electrical Systems and Automation, University of Pisa, Pisa, Italy; 2 Department of Endocrinology and Metabolism, University Hospital of Pisa, Pisa, Italy; 3 Dulbecco Telethon Institute at Department of Endocrinology and Metabolism, University Hospital of Pisa, Pisa, Italy; 4 Department of Psychiatry, Neurobiology, Pharmacology and Biotechnology, School of Medicine, University of Pisa, Pisa, Italy; RAND Corporation, United States of America

## Abstract

**Background:**

Obesity is unanimously regarded as a global epidemic and a major contributing factor to the development of many common illnesses. Laparoscopic Adjustable Gastric Banding (LAGB) is one of the most popular surgical approaches worldwide. Yet, substantial variability in the results and significant rate of failure can be expected, and it is still debated which categories of patients are better suited to this type of bariatric procedure. The aim of this study was to build a statistical model based on both psychological and physical data to predict weight loss in obese patients treated by LAGB, and to provide a valuable instrument for the selection of patients that may benefit from this procedure.

**Methodology/Principal Findings:**

The study population consisted of 172 obese women, with a mean±SD presurgical and postsurgical Body Mass Index (BMI) of 42.5±5.1 and 32.4±4.8 kg/m^2^, respectively. Subjects were administered the comprehensive test of psychopathology Minnesota Multiphasic Personality Inventory-2 (MMPI-2). Main goal of the study was to use presurgical data to predict individual therapeutical outcome in terms of Excess Weight Loss (EWL) after 2 years. Multiple linear regression analysis using the MMPI-2 scores, BMI and age was performed to determine the variables that best predicted the EWL. Based on the selected variables including age, and 3 psychometric scales, Artificial Neural Networks (ANNs) were employed to improve the goodness of prediction. Linear and non linear models were compared in their classification and prediction tasks: non linear model resulted to be better at data fitting (36% vs. 10% variance explained, respectively) and provided more reliable parameters for accuracy and mis-classification rates (70% and 30% vs. 66% and 34%, respectively).

**Conclusions/Significance:**

ANN models can be successfully applied for prediction of weight loss in obese women treated by LAGB. This approach may constitute a valuable tool for selection of the best candidates for surgery, taking advantage of an integrated multidisciplinary approach.

## Introduction

Obesity is unanimously regarded as a global epidemic and a major contributing factor to the development of many common illnesses seen in medical practice. Obesity represents a serious public health concern, reducing life expectancy and raising health care costs. The dramatic increase in the prevalence of obesity is partly related to the fact that conventional therapies have limited efficacy, and the effective management of obesity has consequently become an important clinical focus [1], [2]. Lifestyle interventions can provide a variable degree of weight loss. The key features are adherence to a dietary strategy and exercise programs, but high relapse rates are usually reported. Hope for the development of new anti-obesity drugs grows out of progress that is being made in our understanding of the mechanisms that control body weight and body energy homeostasis. Yet, available pharmacotherapy options are limited and in severely obese subjects their efficacy is usually inadequate and temporary.

The greatest excitement in obesity treatment has come from increasing evidence of the effectiveness of surgical approaches. Recent studies demonstrate a reduction in mortality, beside dramatic benefits in comorbidities, in obese patients treated surgically. In addition, after bariatric surgery, most patients report improvement in psychosocial functioning and quality of life. Altogether, this has lead to an exponential increase in numbers of procedures performed during the last ten years [3]. Surgery is considered the treatment of choice in extreme or morbid obesity (Body Mass Index - BMI≥40). It reverses, ameliorates, or eliminates major cardiovascular risk factors, including diabetes, hypertension, and lipid abnormalities, also when obesity is less severe (BMI≥35). Bariatric surgery should be conducted in centers that are able to assess patients before surgery and to offer a comprehensive approach to diagnosis, treatment, and long-term follow-up. Bariatric surgery includes restrictive procedures as well as procedures limiting the absorption of nutrients. Each of these procedures has its own set of expected outcomes and potential complications. Laparoscopic Adjustable Gastric Banding (LAGB) is one of the most popular restrictive bariatric surgical approaches worldwide. Briefly, a flexible silicone band lined with an inflatable balloon is wrapped around the stomach to create a small upper portion with a narrow opening to a lower large portion. The band is connected to an injection reservoir that is implanted on the abdominal wall underneath the skin, through which the balloon can be inflated or deflated to increase or decrease the restriction. Inflation of the balloon tightens the band and slows down food progression, eventually making patients feel full faster and longer, but this may promote nausea and vomiting. Adjustments are made periodically based on the patient's individual needs.

LAGB has documented satisfactory long-term weight loss, has the best record of safety among the bariatric operations, does not compromise nutrient absorption, is reversible, and can be performed at a relatively low cost. One further advantage lies in long-term adjustability, which can help maximize weight loss while minimizing adverse symptoms [4], [5]. Yet, substantial variability in the results and significant rate of failure can be expected, and it is still debated which categories of patients are better suited to this type of bariatric procedure. In this regard the psychological profile of the candidate patient is thought to be of great relevance. Several studies have been performed to identify potential predictors of success of LAGB, but the existing literature on this matter is far from conclusive. Inconsistent and sometime contradictory results have been reported when BMI, sex, age, physical and psychological factors have been analyzed for their ability to influence the outcome in patients undergoing LAGB [6]–[15]. The reasons for these discrepancies may be related to the peculiar “behavioral” effects of bariatric surgery on obese subjects who are going to lose weight as long as they are able to change their habits after surgery [16]–[20]. A recent French nationwide survey shows that the best profile for a success after gastric banding is a patient <40 years, with an initial BMI<50, willing to change his or her eating habits and to recover or increase his or her physical activity after surgery and who has been operated by a team usually performing >2 bariatric procedures per week [21]. Indeed, patient's ability to fulfill postoperative behavioral changes necessary for success is dependent not only on patient's individual characteristics but also on the experience and skill of the multidisciplinary team that assists the patient during its treatment course and that must provide technical, motivational and psychological support. Therefore, it is not unexpected that predictors of success of LAGB may differ depending on the cultural, social, ethnical or temporal context in which the obesity center is operating.

The effectiveness and the risk-benefit profile of medical intervention require advanced data analysis to classify patient typologies and to predict the effects of therapies in each class. This goal can be set by joining the experience of a medical team, expert in obesity treatments and researchers in the fields of model identification and data mining.

Artificial Neural Networks (ANNs) [22] are flexible non linear mathematical systems capable of modeling complex functions. ANNs can be applied each time there is a relationship between independent predictor variables (inputs) and dependent predicted variables (outputs), even when that relationship is composite, multidimensional and non linear. Another advantage is that ANNs learn by example, and a peculiar outcome (e.g. weight loss) can be associated with an interactive combination of changes on a subset of the variables being monitored (e.g. patients' characteristics) by training algorithms that automatically take into account also the influence of a peculiar environment (obesity center) that mediates the relationship between predictors and outcome. ANNs appear to be better at prediction of weight loss after bariatric surgery than do traditional strategies such as logistic regression [23].

The aim of this study was to investigate the performance of ANN models for prediction of weight loss in obese women treated by LAGB, and to provide an instrument of clinical value in the selection of patients that may benefit from LAGB. Patients' age and BMI were chosen as these parameters have been consistently reported among predictors of LAGB success. Data collected by the Minnesota Multiphasic Personality Inventory-2 (MMPI-2) were employed since this is one of the most common psychometric tests that provides an objective understanding of the motivational patterns as well as a broadband measure of patient's personality and psychopathology.

## Methods

### Participants

From March 2003 to September 2006, 235 obese females underwent LAGB (Swedish Adjustable Gastric Band by Ethicon Endosurgery, Johnson and Johnson, New Brunswick, NJ, USA) at the Obesity Center of the University Hospital of Pisa. LAGB, among various surgical procedures, was chosen according to the following selection criteria: BMI 40 to 60 kg/m^2^ or BMI 35 to 40 kg/m^2^ with serious medical conditions related to obesity. Patients with psychotic disorders, major mood disorders, personality disorders, alcohol or substance abuse, bulimia nervosa or binge eating disorder were excluded from LAGB. None of the patients was taking psychotropic drugs at the time of surgery. For each patient presurgical evaluation included a clinical examination, laboratory and instrumental investigation, a psychological and psychopathological evaluation and an assessment of eating behaviour. Clinical and instrumental examinations of each patient were performed following the Italian guidelines for obesity and each patient was treated according to appropriate protocols for his/her condition. After surgery patients were periodically seen at the Center, and Excess Weight Loss (EWL) was calculated at 2 years follow-up.

The psychological/psychiatric assessment consisted in clinical interviews and administration of MMPI-2. MMPI-2 is the most widely used questionnaire for determining the presence of psychopathology, and it has been carefully investigated and normed [24]–[26]. The questionnaire includes 567 statements and subjects have to answer “true” or “false” according to what is predominantly true or false for them. The test is designed for individuals aged 18 and older. The 1^st^ 370 items are divided into 10 clinical scales and 3 validity scales. This study also used content scales which consist in clusters of items concerning the same psychological dimension and behavioral area. Raw scores from each scale are transformed into standardized T scores: on the clinical and validity scales, a T score of 50 is the estimated population average with a standard deviation of 10. A T score of 65, corresponding to 92^nd^ percentile, appears to be an optimal cut-off point for separating the normative samples from a “clinically interpretable” sample. If the T score of the validity-scales exceeds prefixed thresholds (Lie-scale≥80, Infrequency≥90, and Correction≥80), the possibility exists that the test is not valid.

Among 235 patients, 8 MMPI-2 were considered invalid because more than 30 of the 567 questions remained unanswered. Ten patients did not fill out the tests due to poor Italian language (4 patients) or to a low educational level. Twenty-five patients did not return the psychological test with no specific reasons. Among the remaining 192 women, 2 became pregnant within 2 years after surgery and were not included in the analysis. In 5 patients the band had to be removed because of slippage (1 patient) or uncontrollable vomiting. Six patients did not receive the follow-up visit at 2 years: one moved to a foreign country and five had a follow up visit after 2 years and 6 months. Seven patients preferred to be followed-up at a hospital closer to their home city.

Overall, the study population consisted of 172 obese women, aged 19 to 67 years (mean age ± SD  =  41.7 ± 11.3 years) with a mean ± SD presurgical and postsurgical (24-months after the intervention) BMI of 42.5 ± 5.1 kg/m^2^ and 32.4 ± 4.8 kg/m^2^, respectively. Table 1 shows the main phenotype characteristics of the study group before LAGB intervention.

**Table 1:** Clinical characteristics of the study population before LAGB.

	Mean	SD
Age	41.7	11.3
BMI	42.5	5.1
Waist circumference (cm)	122	13
Hip circumference (cm)	129	11
Waist-to-Hip Ratio (WHR)	0.95	0.12
Total Cholesterol (mg/dl)	203.1	37.7
HDL Cholesterol (mg/dl)	53.3	11.2
LDL Cholesterol (mg/dl)	135	35
Triglycerides (mg/dl)	123	56
Serum glucose (mg/dl)	96.4	19.2

	Subjects	%
Metabolic syndrome (ATPIII)*	98	57%
Post-menopausal	37	37%
Diabetes	26	16%

*Metabolic syndrome was defined according to the Adult Treatment Panel III criteria [43].

### Ethics

Ethics Committee approval was not required since patients identity is not disclosed and data were collected during and according to routinary examination of the patients. Patients did not undergo any treatment or examination specifically devised to collect data employed in this study, and for which their informed consent was necessary.

### Statistical methods

At first, a best-subset algorithm was used to select the most significant predictors of the EWL among the psychological scales, age and BMI before LAGB. Selected variables were used in a standard multiple linear regression model. An ad-hoc ANN was then employed to perform a nonlinear regression using the same variables and the EWL: a specific cost function provided a nonlinear formula to achieve the best correlation between EWL and the selected predictors.

Finally, results obtained by the linear and the nonlinear models were applied in standard prediction and classification tasks, by dividing patients according to quartiles of EWL.

#### Best-subset Algorithm and Multiple Linear Regression Model

A multiple linear regression model [27] based on a best-subset algorithm [28] was determined. All MMPI-2 psychological scales (validity, clinical, content and supplementary scales), pre-operative BMI and age were selected as independent variables for the linear model and EWL at 24 months follow-up was chosen as dependent variable (output). EWL was calculated as follows:
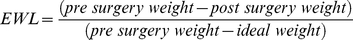
where ideal weight is defined by the Lorentz formula [29] (for female subjects)
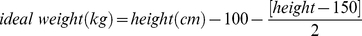
In order to obtain a robust model, only subsets with a number of independent variables ranging from 1 to 4 were calculated. This hypothesis relies on the practical constraint that the size of the data set is limited to 172 patients and the rule of thumb of at least 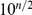 records is considered, where *n* is the number of independent variables included in the regression model. Furthermore the parsimony principle stating that it is preferable to select the model with the smallest numbers of variables was adopted.

All possible combinations of explanatory variables (from one to four) were computed in a multiple linear regression with EWL as the dependent variable, and a list of the values of R^2^, adjusted R^2^, p-value and the standard deviation for each linear model was extracted. Among all models, it was chosen the model with the highest R^2^, the smallest standard deviation and p-value less than 0.05.

R-squared partial correlations were used to measure the marginal contribution of each explanatory variable when all others were already included in the model. Finally, EWL was predicted through a linear combination of regression coefficients *β*.

#### Neural Network Models: Architecture and Learning Algorithm

The ANN [22], [30]–[32] model used in this study was a Multi-Layer Perceptron (MLP), a feed-forward neural network for mapping sets of input data onto a set of appropriate outputs. MLP is characterized by three layers of neurons (input layer, hidden layer and output layer) with nonlinear activation functions at the hidden layer [33].

**Figure 1:**
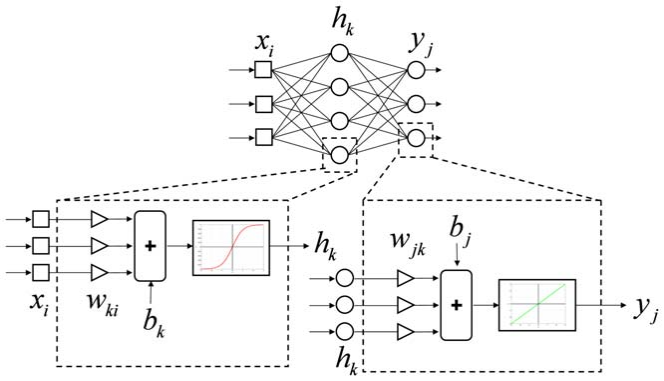
Schematic diagram of multi-layer perceptron. Basic architecture of MLP with one hidden layer of neurons sandwiched between the input layer and the output layer. The hyperbolic tangent function gives non linearity to the entire structure.

The basic architecture of MLP (Fig. 1) consists of an input layer passing input data *x_i_* to a layer of “hidden” neurons with sigmoid activation function, like the hyperbolic tangent function, 
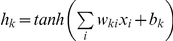
 where 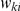 and 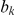 are the weight matrix (between input layer and hidden layer) and bias parameters of hidden layer units, respectively. The outputs *y_j_* of the network are a linear function of the parameters of the last hidden layer 
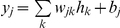
 where 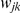 and 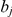 are the weight matrix (between hidden layer and output layer) and bias parameters of output layer units, respectively.

Usually, given observed data 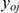, the optimal values for weight and bias parameters (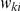, 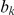, 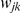 and 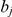) are found by training the MLP, i.e., performing a non linear (due to the use of a non linear function like, e.g., the hyperbolic tangent) optimization for which the mean square error of the output 
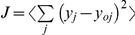
 is minimized. *J* is called the cost function or objective function of MLP. When the training algorithm is stopped, the MLP has found a set of non linear regression relations 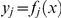.

In this study, in order to identify the best correlation between the independent variables and the EWL, two feed-forward MLPs were used, one for non linear mapping of variables (*x*) into a single score *u*, the other one for linear mapping of EWL (*y*) into a score *v*.

These two networks independently map from the inputs *x* and *y* to the scores *u* and *v*, respectively. A particular cost function forces the correlation between *u* and *v* to be maximized by finding the optimal values of weights and bias.

**Figure 2:**
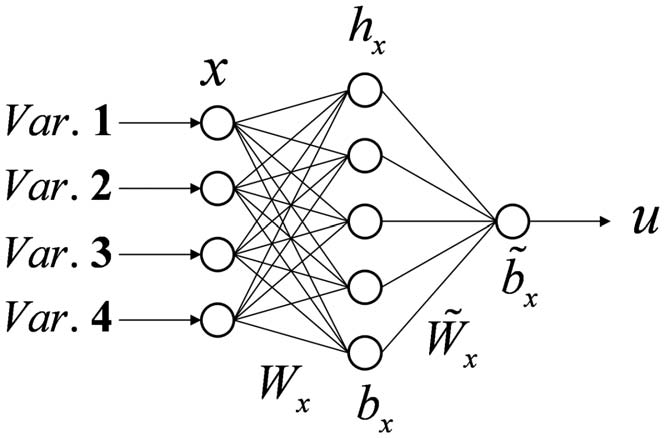
Architecture of the MLP model for calculating non linear score *u*. The MLP maps the four input variables into the non linear score *u* through hidden layer non linear activation functions (i.e., *tanh* function).

In the first MLP (Fig. 2), the input layer consists of variables considered as statistically significant by the previous best-subset algorithm; the hidden layer is characterized by some hidden neurons (in Fig. 2, five hidden neurons), and the output layer consists of one output neuron (the non linear score *u*). For computational issues, input variables were initially standardized by removing the mean value and dividing them by the standard deviation of each variable.

**Figure 3:**
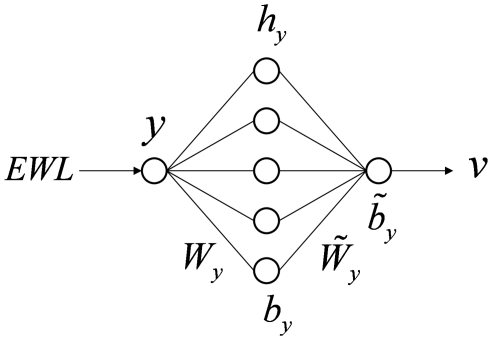
Architecture of the MLP model for calculating linear weight loss score *v*. The MLP maps the EWL into the weight loss score *v* through hidden layer linear activation functions (i.e., identity function).

In the second MLP (Fig. 3), a linear mapping of EWL is performed: this linear mapping (i.e., using a linear activation function) was chosen to simplify the network and to compare the results with those obtained by the linear model based on standard multiple regression. Furthermore, by having a second MLP, a non linear recombination of multiple dependent variables (beside EWL) may be obtained by replacing the linear activation function with a non linear one.

For both MLPs, the number of hidden neurons was determined through a trial-and-error process and following a general principle of parsimony, because no commonly accepted theory exists to determine the optimal number of neurons in the hidden layer: in detail, several runs (i.e., training of MLP) with increasing number of neurons were made. As a result of this step, the number of hidden neurons was chosen when the correlation between 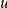 and 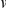 did not improve appreciably by increasing the number of units.

For both MLPs, the input variables vectors 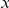 and 
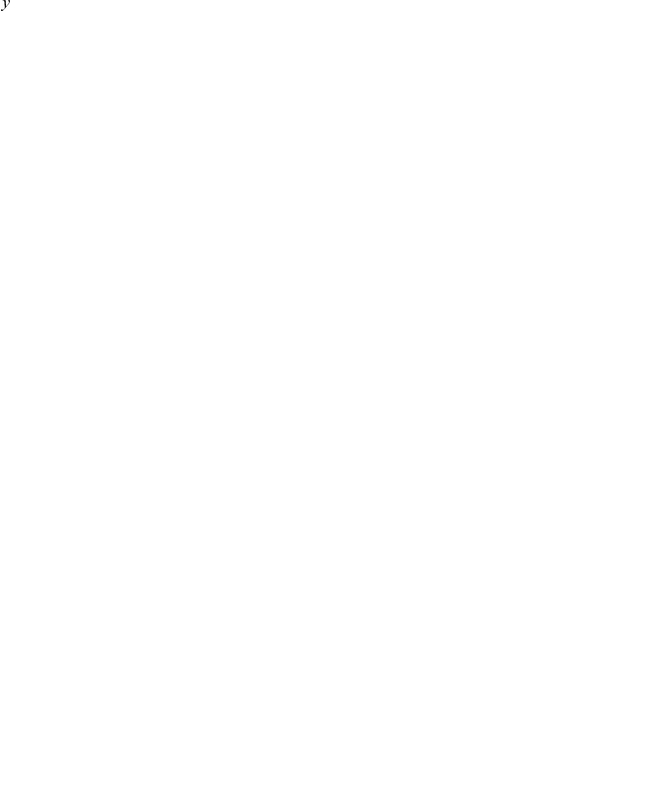
 are mapped to the neurons in the hidden layer 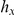 and 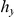 as follows:
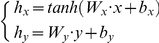
where 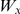 and 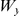 are the weight matrices between input layer and hidden layer and 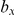 and 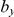 are the bias parameter vectors of hidden layer units. The scores 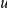 and 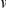 are obtained from a linear combination of the hidden neurons vectors 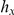 and 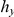, respectively, with
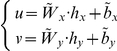
To maximize the correlation between 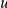 and 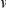, the specific cost function 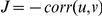 was minimized by finding the optimal weight values and bias between different nodes (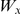, 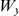, 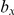, 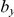, 
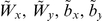
) using all data available. In addition, we applied the constraints 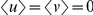 and 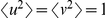 (zero mean and unit variance for both the scores) which were inserted into a modified cost function (*J_m_*):
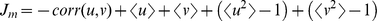
The nonlinear optimization was carried out by a quasi-Newton algorithm. Because of the well-known problem of multiple local minima in the MLP cost function, there was no guarantee that the optimization algorithm reached the global minimum: hence a number of runs (i.e., training of MLP) mapping from 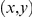 to 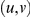 using random initial parameters were performed. The number of runs was fixed to 200 and the run attaining the lowest value of *J_m_* was selected as the final solution.

MLP might suffer from overfitting, i.e., if the MLP has too many parameters, its output will fit very accurately all training set data (including the noise) but it will provide meaningless responses with new data that are not present in the training set. To overcome this pitfall, 20% of the data were randomly selected as validation data and withheld from the training set of the MLP: runs where the correlation between *u* and *v* was found lower for the validation data than for training data set were rejected to avoid overfitted solutions.

#### Classification of Subjects in terms of EWL Outcome

The predictive performance of both models was evaluated by calculation of the true positive fraction (TPF, or sensitivity) and of the false positive fraction (FPF, or specificity). To this purpose patients were divided into 2 groups by using the first quartile of actual EWL as a cut-off value: patients with an EWL within the 3 highest quartiles were arbitrarily assigned to the positive group while patients with an EWL within the lowest quartile were assigned to the negative group. Sensitivity was defined as the rate of patients correctly predicted in the positive group over those actually belonging to the positive group; specificity was defined as the rate of patients correctly predicted in the negative group over those actually belonging to the negative group.

The sensitivity and specificity of both weight scores (obtained from linear and MLP models) in relation to LAGB outcome were plotted for each possible predictive score cutoff in the so-called Receiver Operating Characteristic curves (ROC) and the Area Under each ROC Curve (AUC) was estimated. AUC measures the discriminating accuracy of the (linear or non linear) model, i.e., the ability of the model to correctly classify patients in the positive or in the negative group.

#### Cross-Validation and Prediction

Up to this point both linear and non linear models were built by considering all patients of the data set. In other words, models were built from a database where inputs and output were perfectly known. The following step was to apply the models to new data in order to assess their prediction value by using the cross-validation method and the confusion matrix as analysis tools.

In the cross-validation algorithm, the whole data set is repeatedly split into training and test sets, and data from the test set are classified with the model obtained from the training set. In the case of the non linear model one group of patients was used as test data in order to make a prediction of the EWL (Test Set), and the others were used for training the MLP (Training set).

The same procedure with the same partitions was conducted in the case of the linear model, calculating linear regression coefficients *β* from training data set and making a prediction of the output from the test set.

Therefore in the test phase, each model made a prediction of EWL based only on the test set. If the predicted EWL value belonged to the same quartile of the actual EWL of the patient under test, the prediction was considered correct.

Confusion matrix [34] was used as a tool for evaluating effectiveness of model prediction; this is a table that allows a comparison of the accuracy of the predicted EWL-quartile membership against the actual membership. Each predicted quartile was plotted against the actual one and the number of subjects classified within each quartile gave an indication on the effectiveness of the prediction.

In other words, the model tried to classify patients into four possible classes of EWL, considering the selected variables. The elements of the matrix (its dimension was *4*×*4*) represented the percentage of patients that were correctly classified within each class.

The whole procedure was as follows:

1. The sample was subdivided into three homogeneous random subgroups;
2. Both MLP and linear regression models were trained with two of the three subgroups and the third group was used to test the model: a confusion matrix was calculated from the results of the test operation (i.e. the number of patients properly classified by the model, expressed as percentage);
3. Step 2 was repeated cyclically, exchanging subgroups for training and for testing. From the confusion matrices that were obtained, the mean value of each element was computed to express the global model prediction. This allowed the training algorithm to use virtually the entire data set for training;
4. The cross-validation algorithm was repeated 100 times with different subsets of patients for training and test sets, for both the linear and the non linear models.

All statistical comparisons and analysis (best subset algorithm, multiple linear regression and MLP models, ROC curves and cross validation with confusion matrix) were performed using Matlab™, by a toolbox named “Obefix” [35].

## Results

After LAGB, an average of 48.19% EWL (SD  =  19.71%) was observed. There was a large difference in EWL among patients, ranging from almost complete weight normalization to absence of weight loss (range of EWL 0–91.3%). When patients were divided into quartiles based on EWL achieved by LAGB, the EWL upper thresholds between consecutive quartiles were 35%, 48.9% and 62.8%

The distribution of MMPI-2 scores obtained in our sample of 172 obese subjects before surgery is reported in Tables 2, 3 and 4. MMPI-2 scale scores were categorized in four classes (<50, 50–64, 65–74 and ≥75).

**Table 2:** Percent distribution (and cases) of obese subjects based on MMPI-2 T scores of validity scales as compared with the normative population.

	Normative population		Obese subjects	
T score		L*	F	K
<50	50.00	15.70 (27) **	59.88 (103)	43.02 (74)
50–64	43.32	59.88 (103)	35.47 (61)	44.77 (77)
65–74	6.06	21.51 (37)**	3.49 (6)	12.21 (21)
≥75	0.62	2.91 (5)	1.16 (2)	0.00 (0)

Legend: L - Lie; F - Infrequency; K – Correction.

*Chi-Square *X^2^* test p<0.05 as compared with the normative population,

**residual post-hoc p<0.05 as compared with the normative population.

**Table 3:** Percent distribution (and cases) of obese subjects based on MMPI-2 T scores of clinical scales as compared with the normative population.

	Normative population					Obese subjects					
T score		Hs*	D	Hy	Pd*	Mf	Pa	Pt	Sc*	Ma	Si
<50	50.00	24.42 (42)**	44.77(77)	39.53(68)	8.14(14)**	40.70(70)	47.09(81)	59.88(103)	23.26(40)**	66.28(114)	53.49(92)
50–64	43.32	52.33(90)	48.26(83)	46.51(80)	86.63(149)**	48.26(83)	46.51(80)	37.79(65)	72.09(124)**	30.23(52)	38.95(67)
65–74	6.06	18.02(31)	6.40(11)	12.21(21)	5.23(9)	11.05(19)	6.40(11)	1.16(2)	4.07(7)	2.91(5)	5.81(10)
≥75	0.62	5.23(9)	0.58(11)	1.74(3)	0.00(0)	0.00(0)	0.00(0)	1.16(2)	0.58(1)	0.58(1)	1.74(3)

Legend: Hs – Hypochondriasis; D – Depression; Hy – Hysteria; Pd - Psychopathic Deviate; Mf - Masculinity–Femininity; Pa – Paranoia; Pt – Psychasthenia; Sc – Schizophrenia; Ma – Hypomania; Si - Social Introversion.

*Chi-Square *X* ^2^ test p<0.05 as compared with the normative population,

**post-hoc p<0.05 as compared with the normative population.

**Table 4:** Percent distribution (and cases) of obese subjects based on MMPI-2 T scores of content scales as compared with the normative population.

	Normative population							Obese subjects								
T score		Anx	Frs	Obs*	Dep	Hea	Biz	Ang*	Cyn	Asp	TpA	Lse	Sod	Fam*	WRK	TRT
<50	50.00	44.77(77)	53.49(92)	73.26(126)	58.72(101)	32.56(56)	46.51(80)	73.26(126)	58.14(100)	64.53(111)	62.79(108)	47.67(82)	50.00(86)	68.02(117)	61.05(105)	54.65(94)
50–64	43.32	52.91(91)	37.21(64)	22.67(39)	35.47(61)	52.91(91)	48.84(84)	23.84(41)	33.14(57)	32.56(56)	27.91(48)	45.93(79)	41.28(71)	30.81(53)	36.05(62)	35.47(61)
65–74	6.06	1.74(3)	7.56(13)	3.49(6)	5.23(9)	12.21(21)	4.65(8)	1.74(3)	7.56(13)	2.33(4)	6.40(11)	4.07(7)	6.40(11)	0.58(1)	2.33(4)	8.72(15)
≥75	0.62	0.58(1)	1.74(3)	0.58(1)	0.58(1)	2.33(4)	0.00(0)	1.16(2)	1.16(2)	0.58(1)	2.91(5)	2.33(4)	2.33(4)	0.58(1)	0.58(1)	1.16(2)

Legend: ANX – Anxiety; FRS – Fears; OBS – Obsessiveness; DEP – Depression; HEA - Health Concerns; BIZ - Bizarre Mentation; ANG – Anger; CYN – Cynicism; ASP - Antisocial Practices; TPA - Type A; LSE - Low Self-Esteem; SOD - Social Discomfort; FAM - Family Problems; WRK - Work Interference; TRT - Negative Treatment Indicators.

*Chi-Square *X* ^2^ test p<0.05 as compared with the normative population.

Chi-square test was used to determine whether the distribution of MMPI-2 scale scores in our cohort of obese females differed significantly from that of the Italian normative population [36]. In this regard it should be noted that MMPI-2 T scores have been computed to ensure that in the normative population a T score of a given level has the same percentile value for all scales. As compared with the normative population, in the validity scale “Lie” a significantly lower proportion of obese women scored lower than 50, and a significantly greater proportion scored between 65 and 74 (Table 2).

In addition, a significantly lower proportion of obese women scored lower than 50 in the clinical scales “Hypocondriasis”, “Psychopathic Deviate” and “Schizophrenia”. A significantly higher proportion of obese women fell within category 50–64 in clinical scales “Psychopathic Deviate” and “Schizophrenia” (Table 3).

Regarding the content scales “Obsessiveness”, “Anger” and “Family Problems“, our cohort significantly differed from the normative population, showing predominantly lower scores (Table 4).

### Multiple Linear Regression Model

As a result of best-subset regression algorithm, a model with the independent variables age, “Paranoia” (Pa), “Antisocial Practices” (Asp) and “Type-A Behaviour” (TpA) was selected (Table 5). These four independent variables accounted for about 10% of the weight loss variance: the Pearson coefficient of correlation *r*, coefficient of determination *R^2^* and p-value are shown in Table 6.

**Table 5:** Multiple linear regression coefficients.

	Unstandardized Coefficients		Standardized Coefficients					
	B	Std. Error	Beta	t	Sig.	Zero-order Correlation	Partial Correlation	VIF
(Constant)	96.977	13.932	-	6.961	p<0.001*	-	-	-
**Age**	−0.259	0.128	−0.148	−2.020	0.045*	−0.129	−0.154	1.007
**Pa**	−0.510	0.159	−0.239	−3.211	0.002*	−0.201	−0.241	1.032
**Asp**	−0.626	0.210	−0.260	−2.985	0.003*	−0.122	−0.225	1.418
**TpA**	0.364	0.172	0.182	2.118	0.036*	0.044	0.162	1.384

* = p<0.05.

**Table 6:** Multiple linear regression model summary.

N	R	R Square	Adj. R Square	Std. Error	F	p-value
172	0.326	0.107	0.085	18.85	4.98	p<0.001

Table 5 also illustrates that the Variance Inflation Factor (VIF) values for this model varied between 1.418 for Asp scale and 1.007 for age which are far below the recommended level of VIF  =  5 [37]: therefore, VIF values suggested that independent variables included in this model did not suffer from the problem of multicollinearity.

The analysis of residuals confirmed the validity of the model: they had zero mean, Gaussian distribution (confirmed by statistical tests of Jarque-Bera and Lilliefors) and were independent (hypothesis confirmed by Runs Test).

**Figure 4:**
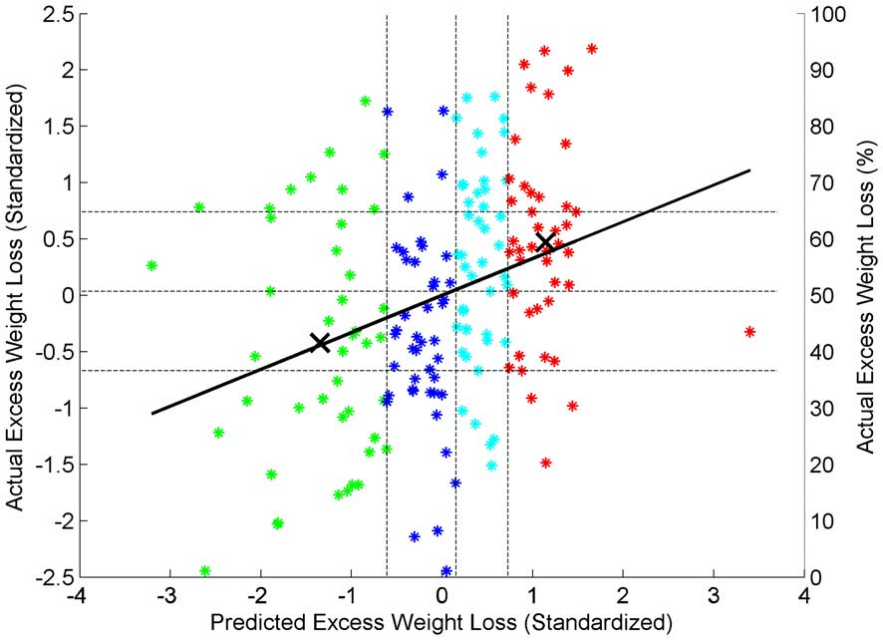
Linear regression model. Figure shows predicted EWL on x-axis versus actual EWL on y-axis. Solid line represents best fit line (r = 0.326), green points are subjects with predicted EWL belonging to the first quartile, blue points to the second quartile, cyan to the third and red to the fourth quartile. Vertical and horizontal dotted lines denote quartiles of predicted EWL and actual EWL, respectively. Black crosses indicate centroids (mean values) of the first and the last quartiles.

A predicted EWL score (standardized) was calculated through the formula 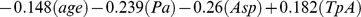. A simple regression analysis was then conducted with actual EWL (standardized) as dependent variable and predicted EWL score (standardized) as the independent variable (Fig. 4). Results are summarized in Table 6.

### Multi-Layer Perceptron Model

As a result of MLP training with increasing number of neurons, the correlation between 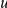 and 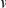 did not improve appreciably by increasing the number of neurons in hidden layer over five. Therefore, the number of hidden neurons was fixed to 4 for both MLPs.

**Figure 5:**
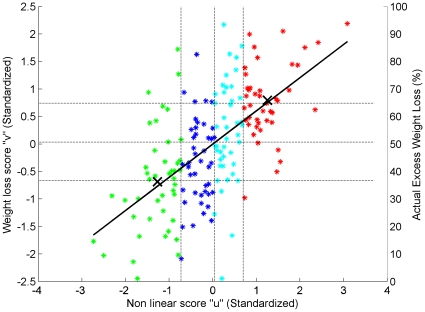
Non linear regression model. Figure shows non linear score *u* on x-axis versus EWL score *v* on y-axis. Solid line represents best fit line (r = 0.604), green points are subjects who have u score belonging to the first quartile, blue points to the second quartile, cyan to the third and red to the fourth quartile. Vertical and horizontal dotted lines denote quartiles of *u* and *v* score, respectively. Black crosses indicate centroids (mean values) of the first and the last quartiles.

When the same input/output data were put as input of MLPs, the Pearson correlation coefficient between nonlinear score *u* and weight loss score *v*, was 0.604, significantly greater than that obtained with the linear model. In addition, R^2^ increased from 0.1 to 0.365 and the standard error of estimate decreased from 0.948 to 0.799 which indicated a better fit for the non linear model (Table 7 and Fig. 5).

**Table 7:** Linear (multiple regression), all 2-way interactions model and non linear (MLP) models summary.

MODEL	N	R	R Square	Std. Error	F	p-value
Linear	172	0.326	0.107	0.948	20.18	p<0.001
All 2-way interactions model	172	0.518	0.268	0.823	50.72	p<0.001
Non linear	172	0.604	0.365	0.799	97.55	p<0.001

Furthermore, in order to validate the performance of the non linear model, a multiple regression model with all two-way interactions between variables significantly correlated with EWL, was computed. Seven variables (main effects) and their interactions accounted for 26.8% of EWL variability, greater than the linear model (R^2^  =  10%) but lower than the nonlinear model which explains 36.5% variability (Table 7).

### Comparison between Linear and Non Linear Models

A quartile division of linear and non linear scores was performed in order to identify 4 classes of predicted EWL for both models, on the basis of the values of age and selected psychological variables (Fig. 4–5).

Centroids were calculated for the first and the fourth quartiles, which resulted in a EWL interval of 20% in the case of the linear model whereas the interval increased to over 30%.

The nonlinear model allowed a better separation among quartiles and better overlapping between predicted EWL and actual EWL with respect to the linear model (Tables 8–9, Fig. 4–5).

**Table 8:** Distribution of obese subjects according to the linear regression model, based upon quartile division of predicted (columns) and actual (rows) weight loss.

Actual \ Predicted EWL quartiles	1°	2°	3°	4°	Total
**4°**	9 (20.9%)	4 (9.3%)	15 (34.9%)	15 (34.9%)	43 (25.0%)
**3°**	6 (14.0%)	10 (23.3%)	12 (27.9%)	15 (34.9%)	43 (25.0%)
**2°**	9 (20.9%)	14 (32.6%)	11 (25.6%)	9 (20.9%)	43 (25.0%)
**1°**	19 (44.2%)	15 (34.9%)	5 (11.6%)	4 (9.3%)	43 (25.0%)
**Total** *	43 (100.0%)	43 (100.0%)	43 (100.0%)	43 (100.0%)	172 (100.0%)

*100.0%  =  total number of subjects for each quartile of predicted EWL.

**Table 9:** Distribution of obese subjects according to the non linear regression model, based upon quartile division of predicted (columns) and actual (rows) weight loss.

Actual \ Predicted EWL quartiles	1°	2°	3°	4°	Total
**4°**	3 (7.0%)	5 (11.6%)	14 (32.6%)	21 (48.8%)	43 (25.0%)
**3°**	6 (14.0%)	11 (25.6%)	9 (20.9%)	17 (39.5%)	43 (25.0%)
**2°**	13 (30.2%)	11 (25.6%)	15 (34.9%)	4 (9.3%)	43 (25.0%)
**1°**	21 (48.8%)	16 (37.2%)	5 (11.6%)	1 (2.3%)	43 (25.0%)
**Total** *	43 (100.0%)	43 (100.0%)	43 (100.0%)	43 (100.0%)	172 (100.0%)

*100%  =  total number of subjects for each quartile of predicted EWL.

This held true both for subjects in the upper quartile (mean predicted EWL by linear model  =  57%, mean predicted EWL by non linear model  =  63.8%, mean actual EWL  =  72.9%) and for subjects in the lower quartile (mean predicted EWL by linear model  =  40%, mean predicted EWL by non linear model  =  34.7%, mean actual EWL  =  22.7%).

### ROC Curves

**Figure 6:**
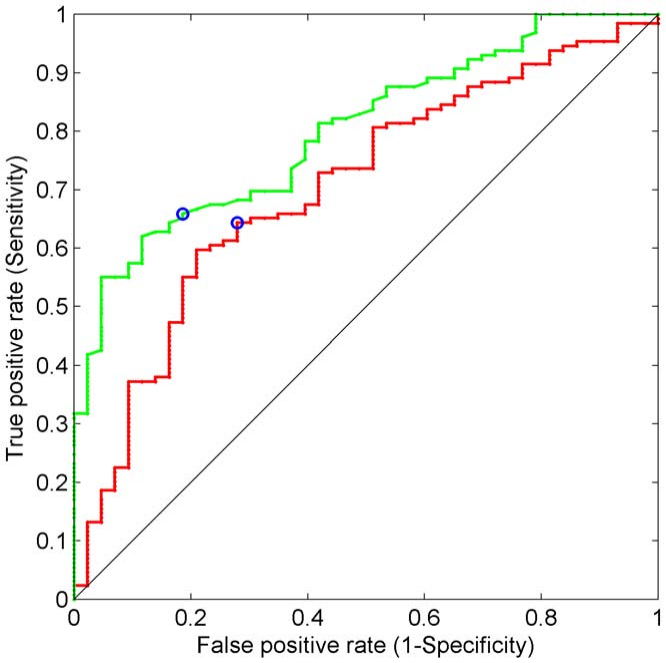
ROC curves for LAGB outcome classification model. ROC curves for both linear and non linear models (see Methods). Sensitivity, or true positive rate, is plotted on the y-axis, and false positive rate, or 1 minus specificity, on the x-axis. Solid green, red and black lines represent non linear, linear model and random classifier, respectively. Blue circles represent the best cut-off values for both models calculated as the closest point of each curve to the upper left corner.

Sensitivity and specificity in predicting LAGB outcome were determined from ROC curves based on predicted EWL scores (Fig. 6). Roc curves were built by dividing patients into two groups using the first quartile as a threshold.

As for the linear model, the best cutoff point (i.e., the closest points to the upper left corner) of predicted EWL (standardized) was −0.0024 (true positive  =  83; false positive  =  12; true negative  =  31 and false negative  =  46). This cut off point corresponds to 50.1% of EWL. Accuracy and mis-classification rate were 66.3% and 33.7%, respectively (Tables 10 and 11).

**Table 10:** Results of ROC analysis.

				Asymptotic 95% Confidence Interval	Asymptotic 95% Confidence Interval
	AUC	Std. Error	Asymptotic Sig.	Lower Bound	Upper Bound
**LINEAR MODEL**	0.704	0.045	0.000	0.616	0.792
**NON LINEAR MODEL**	0.801	0.035	0.000	0.734	0.869

**Table 11:** Performance of linear and non linear model classifiers at best cutoff points.

Model	Prevalence	Sensitivity	False Negative Rate	Specificity	False Positive Rate	Accuracy	Mis-classification Rate
**LINEAR**	75.0%	64.3%	35.7%	72.1%	27.9%	66.3%	33.7%
**NON LINEAR**	75.0%	65.9%	34.1%	81.4%	18.6%	69.8%	30.2%

As for the non linear model, the best *u* cutoff point was −0.09 (true positive  =  85; false positive  =  8; true negative  =  35 and false negative  =  44). This cut off point corresponds to 49.9% of EWL. Accuracy and mis-classification rate were 69.8% and 30.2%, respectively (Table 10 and 11).

### Prediction

The cross-validation algorithm was used to extend the prevision capability of our models to new data. Results of the cross-validation algorithm are reported as the average of 100 confusion matrices (Tables 12 and 13). By using the linear model, 63% patients (40% + 23%) with a predicted EWL within the 1^st^ quartile achieved an actual EWL <48.9% (i.e., the median value of actual EWL). At the same time 67% patients (35% + 32%) with a predicted EWL within the 4^th^ quartile obtained an actual weight loss of >48.9%. At variance, by using the non linear model, the proportion of patients correctly predicted below or above the median value of actual EWL raised up to 70% (29% + 41%) and 78% (45% + 33%) for the 1^st^ and the 4^th^ quartiles, respectively. By both models a poor prediction was obtained when patients fell within the 2^nd^ or the 3^rd^ quartiles of predicted EWL.

**Table 12:** Prediction value (mean ± SEM) of the confusion matrix obtained by the linear model.

Actual \ Predicted EWL quartiles	1°	2°	3°	4°
**4°**	16 ± 0.5%	9 ± 0.5%	34 ± 0.7%	35 ± 0.4%
**3°**	21 ± 0.6%	23 ± 0.9%	28 ± 0.9%	32 ± 0.6%
**2°**	23 ± 0.7%	33 ± 0.8%	26 ± 0.8%	23 ± 0.5%
**1°**	40 ± 0.5%	35 ± 0.4%	12 ± 0.5%	10 ± 0.5%
Total	100%	100%	100%	100%

**Table 13:** Prediction value (mean ± SEM) of the confusion matrix obtained by the non linear model.

Actual \ Predicted EWL quartiles	1°	2°	3°	4°
**4°**	10 ± 0.3%	20 ± 0.5%	24 ± 0.6%	45 ± 0.3%
**3°**	20 ± 0.5%	23 ± 0.5%	29 ± 0.6%	33 ± 0.5%
**2°**	29 ± 0.4%	30 ± 0.6%	29 ± 0.7%	11 ± 0.4%
**1°**	41 ± 0.4%	27 ± 0.5%	18 ± 0.5%	11 ± 0.4%
Total	100%	100%	100%	100%

## Discussion

This study indicates that elaboration of MMPI-2 scores by ANNs can facilitate weight loss prediction in obese candidates to adjustable gastric banding.

Weight loss after bariatric surgery depends on the ability to produce a permanent reduction of daily food intake, as compared with the amount that caused the development of obesity. However, the expected reduction in caloric intake obtained by restrictive surgery procedures does not invariably lead to predictable long term results. This can be related to adherence to a permanent dietary restriction and lifestyle modification. Predictive factors of adherence are not established in the literature. In this regard MMPI-2 psychological scales represent a potential tool for predicting the success of surgical procedures [38].

To investigate this possibility, in this study the MMPI-2 scores obtained before surgery were correlated to the long term results of weight loss after gastric banding. Patients derived by a preselected sample that, based upon current knowledge, had a high probability of success by this surgical procedure. In particular, patients with high level of psychopathology were preliminarily excluded from LAGB. Indeed, results of MMPI-2 don't show a prevalence of psychopathology in this obese sample, which is in excess of the population norms. Yet, our population reported higher scores in validity scale “Lie” that may reflect an unsophisticated defensiveness in which respondents are denying negative characteristics and claiming positive ones because they judge it to be in their best interest [26]. Higher scores in the clinical scale Hypocondriasis are probably related to real physical problems and a psychological component to the illness should be suspected. Similarly, the higher prevalence of high scores in the Psychopathic Deviate clinical scale may indicate the search for immediate gratification of impulses and a limited frustration tolerance. Furthermore, higher frequency of scores than the population expectancy on clinical scale Schizophrenia suggests that patients feel insecure, inferior, incompetent and dissatisfied to their life situation. These results should be interpreted in light of some intrinsic limitations. First, the psychopathologic profile of our sample belongs to individuals seeking bariatric surgery, and cannot be generalized to all obese subjects dealing with a medical condition. Second, as already mentioned, patients were selected to meet criteria that, based on our own experience and on that derived from the literature, are associated with the best probability of long-lasting weight loss after gastric banding. This is why our data are not aligned with previous studies that concern either the general population of obese subjects or unselected obese candidates for bariatric surgery, which show higher level of psychopathology, in particular on scales regarding anxious-depressive symptoms [39], [40].

On average, weight loss observed in our study group at 2-years follow-up was in line with that reported in the literature, to indicate that our selection criteria complied with the international guidelines for gastric banding. However, as expected, there was a great variability among subjects. The best subset algorithm highlighted the variables “age”, “Pa” (Paranoia), “Asp” (Antisocial Practices), “TpA” (Type-A Behavior) as significant predictors of EWL. According to Busetto et al. [41] the weight loss achieved by LAGB in older patients is lower (but it is still associated with a significant improvement in comorbidities). Similarly, Singhal et al. [42] reported a higher, though not significant, EWL in patients with age less than 50 years. The clinical scale 6 of MMPI-2 (Paranoia) consists in 40 items. Some of those items deal with frankly psychotic behavior (suspiciousness, ideas of references, delusions of persecution and grandiosity). Others items cover such diverse topics as sensitivity, cynicism, asocial behavior, excessive moral virtue and complaints about other people. It is possible to obtain a T score greater than 65 on this scale without endorsing any of the frankly psychotic items. The content scale “Antisocial Practices” (Asp) consists in antisocial attitudes and antisocial behavior. The content scale “Type-A (TpA) consists in impatience and in competitive drive [26].

In our study, age, paranoia and antisocial practices showed an inverse correlation with EWL while Type-A Behavior had a positive correlation with it. Overall, these four independent variables accounted for 10% of the weight loss variance, which is significant but of very limited value in the clinical practice.

When the MLP model was applied, the weight loss variance predicted by the 4 variables raised up to 36%, with accuracy and mis-classification rates of 70% and 30%, respectively. As patients were selected to exclude those with high levels of psychopathology, the inputs variables generated by MMPI-2 spanned over a relatively limited range of scores. We might speculate that if non-selected patients had to be included in the study, a greater variability of MMPI-2 scores would have been obtained and the prediction value of our model might have been even greater. At present, we believe that this model is the best available tool that objectively exploits psychological scores in the selection of candidates for gastric banding.

Our ANN approach extends the predictive range of the linear regression model, by replacing the identity functions with nonlinear activation functions, and it appears more suitable to describe complicated systems. ANNs may be trained with data gained in various clinical contexts, to take into account local expertise, racial differences as well as other unknown variables that can affect the clinical outcome. The analysis may not be necessarily limited to psychological parameters and other potentially useful variables could be tested to improve the predictive value of the model. Furthermore, our ANN architecture using 2 MLPs is potentially able to include more than one dependent variable (in addition to EWL) and operate a non-linear transformation between them. Future research using biochemical or anthropometric variables may build on these observations.

In conclusion, results of this study, validated in random samples of the same population, demonstrate that it is possible to establish with over 70% of reliability what the final outcome of the intervention will be in those individuals that will either maximally or minimally benefit from LAGB. In practical terms this innovative approach, totally non invasive, may constitute a precious tool to establish which are the best candidates to the interventions and reduce costs, sufferance and failure to those that wouldn't comply sufficiently to the therapy.

### Limitations

One of the main drawbacks of ANN approach is the impossibility to discriminate what is the real contribution of each variable in the final prediction: ANN is a good technique to perform predictions if lot of data are available to train the algorithm but at the cost of loss of power of explanation.

A further limitation of ANNs is that, due to local minima in the cost function, optimizations starting from different initial parameters, often ends up at different minima. Therefore, a number of optimization runs starting from different random initial parameters is needed, and the best run is chosen as the solution even if there is no guarantee that the global minimum of the cost function has been found.

In addition, the number of hidden neurons in the ANNs is determined by a trial-and-error approach. Adopting techniques such as generalized cross validation and information criteria may help in the future to provide more guidance on the choice of the most appropriate ANN architecture.
